# Unwitnessed magnet ingestion in a 5 year-old boy leading to bowel perforation after magnetic resonance imaging: case report of a rare but potentially detrimental complication

**DOI:** 10.1186/1754-9493-6-16

**Published:** 2012-07-19

**Authors:** James R Bailey, Eric A Eisner, Eric W Edmonds

**Affiliations:** 1 Department of Orthopaedic Surgery, Naval Medical Center, 34800 Bob Wilson Drive, San Diego, CA, 92134, USA; 2 Department of Orthopedics/Scoliosis/Sports Medicine, Rady Children’s Hospital, 3030 Children’s Way, Ste 410, San Diego, CA 92123, USA; 3 Department of Orthopaedic Surgery, University of California, San Diego, UCSD Medical Center, Hillcrest, 200 West Arbor Drive, San Diego, CA 92103, USA

## Abstract

**Background:**

The ingestion of non-food items in children is a relatively common event, often unwitnessed, unknown, and unreported. For those children brought in for medical evaluation, less than 10% require intervention, and only 1% require surgery. This, however, is not the case for magnet ingestion. Magnets, in plurality, can become attracted to one another through intestinal walls, causing a variety of surgical emergencies.

**Case presentation:**

We present a case of unwitnessed multiple magnet ingestion in a 5 year-old boy who presented to the emergency department with the atypical chief complaint of neck pain. The diagnostic work-up including a neck magnetic resonance imaging (MRI) potentially led to bowel perforations managed definitely by a subsequent exploratory laparotomy. The child had an uneventful postoperative recovery and was discharged to home upon surgical recovery.

**Conclusions:**

Institutions should make all possible efforts to attempt to prevent such potential life-threatening circumstances. We propose a screening tool that can further enhance the care of children who cannot or do not report unwitnessed magnetic ingestion prior to MRI evaluation.

## Introduction

Children are prone to ingest many non-food objects that will often pass uneventfully through their digestive system. Magnets in plurality, however, are often attracted to each other even through intestinal walls and multiple reports of pressure necrosis, bowel perforation, volvulus, intestinal fistulas, and obstruction have been documented in the literature [1-4]. With these grim consequences, many pediatric surgeons view magnet ingestion as one of the few non-food ingestions that mandate surgical retrieval – independent of concerning signs, symptoms, or physical exam findings [4-7]. We present a case of unwitnessed magnet ingestion, potentially complicated by magnetic resonance imaging as part of the diagnostic evaluation.

## Case presentation

A five-year-old boy presented to the emergency department with neck pain. Per the parent’s report, the child had been unattended in a room in the home watching television when he began to complain of acute onset posterior neck pain. There was no reported history of trauma. There was no history of fever, sore throat, or headache. The child was administered acetaminophen orally, with no relief of symptoms. The child was then brought to the emergency department, where a thorough evaluation was performed due to continued pain and refusal to move the neck. The past medical history was significant for right-sided congenital muscular torticollis, bilateral developmental dysplasia of the hip, and mild gross and fine motor delay. The torticollis was treated with physical therapy and had resolved prior to this visit. The child’s hip dysplasia was treated with a Pavlik harness and had significantly improved at the time of the most recent radiographic examination, one year prior.

Initial evaluation revealed a healthy-appearing child in no acute distress. His vital signs were stable. Examination of the mouth showed the oropharynx to be clear. Eye exam was normal. Abdominal examination was normal. The child had focal tenderness to palpation over the midline of the entire cervical spine. He was noted to resist range of motion of the neck in all directions, but there was no asymmetry of motion noted. Neurologic examination of the cranial nerves and extremities was normal.

Laboratory studies, including C-reactive protein, were normal. CT scan of the neck was normal. Lumbar puncture was performed in the emergency department and was negative. Throat culture was negative. The child received anti-inflammatory medication and narcotics in the emergency department without resolution of the neck pain. The decision was made by the Pediatric service to admit the child to the hospital and obtain a magnetic resonance imaging (MRI) scan (1.5 Tesla) under moderate conscious sedation, given the child’s young age. MRI of the brain and cervical spine was performed and the patient was kept in the inpatient ward for pain control and observation. The MRI was reported as normal that evening. Orthopaedics was then consulted for further evaluation. The child was placed in a soft collar for comfort overnight.

**Figure 1:**
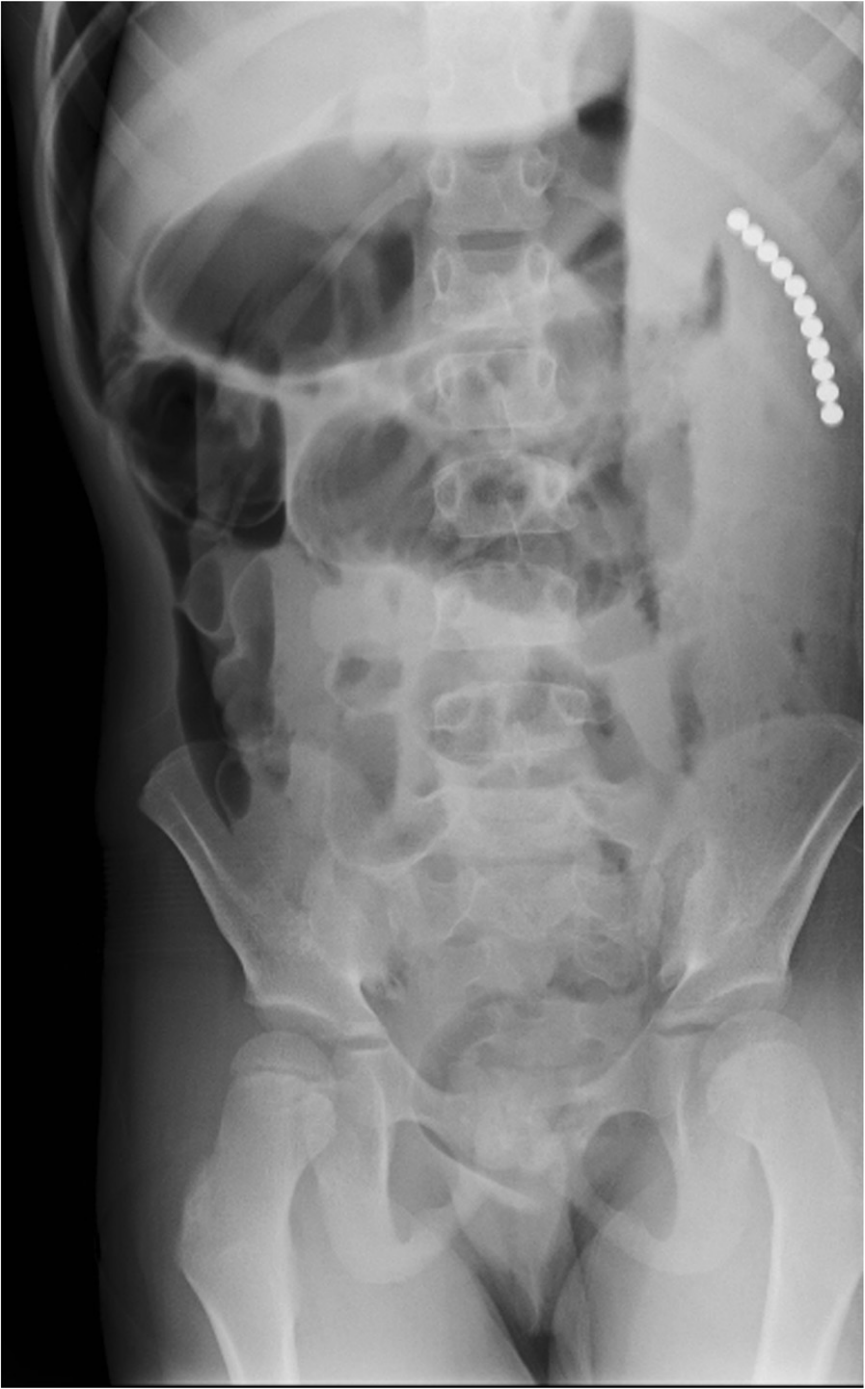
**Left lateral decubitus radiograph obtained in a 5 year-old boy with the new complaint of abdominal pain the morning after an MRI of neck was performed for complaint of neck pain. **Radiograph demonstrates 11 round metallic objects 4 mm in diameter and pneumoperitoneum.

The following morning, the child’s neck pain was noted to be improving, but he was now complaining of severe abdominal pain and refusal to eat. Plain radiographs of the abdomen were obtained which demonstrated pneumoperitoneum and 11 small round metallic objects in the left upper quadrant of the abdomen (Figure 1). The pediatric general surgeon was consulted, and the child was taken to the operating room emergently for exploratory laparotomy. Intra-operatively, four 5–7 mm full thickness perforations to the small intestine were identified and primarily repaired. Eleven small spherical magnets measuring 4 mm in diameter were retrieved from within the peritoneum.

Post-operatively, the child was taken to the pediatric inpatient ward for standard post-operative care. Further questioning of the parents and the child’s sibling on the hospital ward revealed that the child had been playing unsupervised near a magnetic game, and that the child may have ingested some of the small magnetic spheres. His diet was advanced as his bowel function returned over the next several days. His neck pain was noted to still be present, but improving, and had completely resolved by post-operative day five. The child was discharged home on post-operative day number eight and has been doing well since the time of discharge. Ultimately, the child’s neck pain was attributed to irritation of the throat secondary to foreign body ingestion.

## Discussion

The ingestion of non-food items in children is a relatively common event. Most children are asymptomatic, and the event goes without witness. Of those confirmed, less than 10% require intervention, and only approximately 1% require surgery [3,4,6,8-10]. However, ingestion of multiple magnetic objects is potentially very serious and the subject of increased medical scrutiny. The ramifications of magnet ingestion are well documented in the medical literature, primarily as case reports. Cases usually involve young children but have been documented in children up to their teens [1,2,11-13]. All of the documented cases found within the literature presented with either known ingestion or the child’s chief complaint of stomach pain, nausea, and/or vomiting [1-5,14,15]. Many toy products, which incorporate these small but powerful rare-earth magnets, are marketed to young children as building blocks and play pieces.

In 2006, the Center of Disease Control and Prevention (CDC) published in their Morbidity & Mortality Weekly Report a review of three select cases summarizing the 20 known cases of magnet ingestion identified by the Consumer Product Safety Commission (CPSC) from 2003–2006. Of these 20 cases, one resulted in death and the other 19 required gastrointestinal surgery. Ages of children ranged from 10 months to 11 years, 6 months (mean 5 years, 6 months). Boys accounted for 80% of cases. Two of the three cases outlined in this review presented with the chief complaint of stomach pain and vomiting. The third presented after the child self-reported swallowing the building block toy to his mother. At the time of initial presentation and after radiographic confirmation, it was believed that the child had only swallowed one magnet. The family was reassured this object should pass uneventfully. However, even after the same child represented a few days later having also swallowed a small metal ball, this family was again told to monitor stools, as the objects should pass. The following day, the mother read about a death following magnet ingestion, a specialist was consulted, and two 10 mm disc-shaped magnets and a 10 mm steel ball was removed from the small intestines and the affected bowel was resected. This review highlighted a potential grave consequence of magnet ingestion [2].

In 2009, Avolio and Martucciello published a radiographic case report in the New England Journal of Medicine highlighting the cases of a nine-year-old boy who ingested 23 magnets and a 13-year-old developmentally delayed boy who ingested 15 magnets. The nine-year-old child presented four days after ingestion with clinical signs of intestinal perforation and peritonitis while the 13-year-old presented 10 days after ingestion with volvulus and intestinal occlusion. Both were taken to surgery for open exploration and removal. The authors recommended early intervention following magnet ingestion [1].

Others believe that multiple magnet ingestion can be safely, but closely observed and that surgical exploration should be performed only if the child begins to demonstrate these worrisome findings or, if serial radiographic examinations show an unchanged position of two or more magnets side-by-side [4,16,17]. If the magnets remain within the stomach and have not passed through the pylorus sphincter, then some authors advocate for endoscopic removal [6,16,17].

Another often agreed upon criteria for nonsurgical management of magnet ingestion is solitary magnet consumption. Many physicians feel that a solitary magnet poses very little threat to the child and can be allowed to pass spontaneously; however, this practice could prove devastating. On plane radiographs, it can be difficult to discern if the magnet is truly solitary or stuck to multiple magnets in series. A case of bowel perforation from a presumed solitary magnet treated non-surgically at the Mayo Clinic has been documented, and the authors warn against this practice [15].

In 2009, Siddaiah-Subramanya and Borzi presented three cases of magnet ingestion. All three of their cases presented with the initial chief complaint of abdominal pain and vomiting. The history of magnet ingestion was not obtained, so radiographs were not initially ordered and the children were treated symptomatically for gastroenteritis. The first case detailed an 11-year-old autistic child who required laparotomy to remove multiple magnets in adjacent loops of small bowel. This child had areas of small bowel necrosis and 13 perforations. The second case was a four-year-old child who had swallowed multiple magnetic rings. Laparoscopy and subsequent laparotomy were used to repair the small bowel and caecum perforations. The final case was five years old, and laparotomy revealed a perforation of the jejunum secondary to pressure necrosis from multiple magnets. The authors recommended more stringent regulations on the use of magnets in toys, especially for those younger than five years old, and increased awareness in medical providers of the harmful consequences of magnetic toy ingestion [3].

Our patient did not present initially with stomach pain and vomiting but with the chief complaint of neck pain. This initial presenting complaint is not documented elsewhere in the literature. Possibly, this pain was secondary to the magnets passing through the esophagus, and stomach pain and vomiting would have presented in the following few days. The differential diagnosis for isolated neck pain in the pediatric patient is broad, with possible causes ranging from acute trauma to infectious etiologies. Therefore, the work-up of these patients is often extensive and may include advanced imaging studies, such as MRI. Children, under the age of eight years often require a general anesthetic for these studies, thereby disabling their ability to alert providers of an evolving injury during the study. To our knowledge, the case presented here is the first to document bowel perforation following magnetic resonance imaging. Although we cannot definitely identify the MRI as the causative agent for bowel perforation, this case may represent a rare complication of advanced imaging studies and should draw attention to the possibility of complications resulting from unwitnessed foreign body ingestion.

## Conclusion

The differential diagnosis of acute onset neck pain in a child is large, and the workup often involves advanced imaging studies such as MRIs. This case potentially highlights a rare but potential life-threatening complication of magnetic resonance imaging. Health care providers treating children with vague pains must be aware of this potential complication and take all steps necessary to identify an unknown ingested foreign body that can potentially react with the MRI equipment.

Previously at our institution^2^, a thorough written and verbal screening was performed on all children prior to entering the MRI examination room, and a written ‘Patient History Questionnaire’ was completed by the parents. This questionnaire specifically addressed the presence of any implanted metallic devices, including cardiac stents, defibrillators, and orthopaedic implants. Following administration of the written questionnaire, a verbal screening was performed by the technician, addressing any concerning answers on the written survey.

Following this event, a new screening protocol has been instituted. Children and their parents are still asked to complete the written and verbal screenings. In addition, all children are now required to change into a hospital gown and are then screened using a hand-held Ferromagnetic detection scanner (Mednovus SafeScan, Leucadia, CA). Testing at our institution has shown that this ferromagnetic screening tool is able to identify small magnets commonly used in children’s games and toys in both living and cadaveric models. This additional safety precaution has been presented in hopes it will be replicated by other institutions.

## Consent

A release of information with obtained and signed by the patient’s mother.

## Competing interests

The authors declare that they have no competing interests.

The views expressed in this article are those of the authors and do not necessarily reflect the official policy or position of the Department of the Navy, Department of Defense, or the United States Government.

## Authors’ contributions

JRB and EAE were involved in the care of this patient. JRB, EAE, and EWE designed the case report. JRB drafted the first version of the manuscript. EAE and EWE critically revised this paper. All authors contributed and approved the final version of the manuscript.
